# BMT: A Cross-Validated ThinPrep Pap Cervical Cytology Dataset for Machine Learning Model Training and Validation

**DOI:** 10.1038/s41597-024-04328-3

**Published:** 2024-12-28

**Authors:** E. Celeste Welch, Chenhao Lu, C. James Sung, Cunxian Zhang, Anubhav Tripathi, Joyce Ou

**Affiliations:** 1 Center for Biomedical Engineering, School of Engineering, Brown University, Providence, RI 02912 USA; 2 Department of Computer Science, Brown University, Providence, RI 02912 USA; 3 Department of Pathology and Laboratory Medicine, Alpert Medical School, Brown University, Providence, RI 02912 USA

**Keywords:** Cervical cancer, Cancer screening, Biomedical engineering, Scientific data, Computer science

## Abstract

In the past several years, a few cervical Pap smear datasets have been published for use in clinical training. However, most publicly available datasets consist of pre-segmented single cell images, contain on-image annotations that must be manually edited out, or are prepared using the conventional Pap smear method. Multicellular liquid Pap image datasets are a more accurate reflection of current cervical screening techniques. While a multicellular liquid SurePath™ dataset has been created, machine learning models struggle to classify a test image set when it is prepared differently from the training set due to visual differences. Therefore, this dataset of multicellular Pap smear images prepared with the more common ThinPrep® protocol is presented as a helpful resource for training and testing artificial intelligence models, particularly for future application in cervical dysplasia diagnosis. The “Brown Multicellular ThinPrep” (BMT) dataset is the first publicly available multicellular ThinPrep® dataset, consisting of 600 clinically vetted images collected from 180 Pap smear slides from 180 patients, classified into three key diagnostic categories.

## Background & Summary

The 2020 global cancer survey indicated that 604,127 women received a cervical cancer diagnosis that year, while 341,831 died from cervical cancer^1,2^. Over the last several decades, cancer screening using the “Pap smear” approach has helped to catch precancerous abnormalities and increase survival rates^3^.

There has also been a shift in recent years to sample collection using a minimally invasive approach to limit pain by collecting cervical cells with a broom or brush^4^. Furthermore, liquid Pap preparations are now ubiquitously used on a global scale, as they enable improved visualization of individual cells and their features^5–7^.

Along with these advances in sample collection and slide preparation, there is a growing interest in using machine learning technology to automate and advance image processing and classification^8–20^. Using machine learning approaches would help to reduce the burden on cytopathologists and cytotechnologists, who must manually examine tens of thousands of cells for each slide to make diagnoses. This approach would also make diagnosis more feasible in resource limited areas where both physicians and technologists are few and far between.

While significant work has been conducted and published on machine learning approaches for cervical Pap classification, only a select few datasets used for training and testing are publicly available^21^. Furthermore, datasets can be limited in certain ways that impact machine learning classification of liquid Pap smears.

For example, some popular databases consist of images captured from conventional, non-liquid Pap preparations, which are not an adequate training match for liquid Pap smear machine learning workflows due to significant visual differences. Examples include the Center for Recognition and Inspection of Cells CRIC Cervix collection, consisting of 400 images with individual cell classifications^22^.

Different datasets use various staining approaches, including H&E staining and Papanicolau stain^23^. Other publicly accessible datasets with liquid-based Pap images use SurePath™ or cytospin preparations, which are visually distinct from ThinPrep®^24,25^. Some datasets feature annotations that are made directly on images (e.g. lines, circles, arrows, etc.) for training purposes. In this case, images would need further manual processing (cropping and re-evaluation) prior to training.

There are datasets that consist solely of individual pre-segmented cells, such as the popular Herlev conventional Pap dataset, consisting of 70–197 individual cells per class^26^. This may be useful for certain applications; however, if the intent is to create a multicellular classification schematic, or even to incorporate auto-segmentation, these pre-segmented images will not be relevant or useful in model training.

Another issue present in several clinical datasets is class imbalance, which can affect machine learning training and model performance. Most datasets are heavily skewed towards the benign “negative for intraepithelial lesion or malignancy” (NILM) class, for example, as there are simply more available patient slides with this classification. In the Hussain *et al*. dataset, for example, 64% of the 963 total images are from this class, while other pathologically relevant classes are represented in smaller percentages^27^.

Our previous machine learning work has illuminated the importance of training machine learning models on datasets that closely resemble clinical test images^28^. Differences in the staining, preparation, and image capture technique introduced significant variability that prevented a model trained on the Hussain *et al*. SurePath™ Pap image database from easily classifying ThinPrep® images. Consistency in staining, preparation, magnification, and other metrics are essential to create databases that are clinically relevant.

Given the expanding use of ThinPrep® Pap smears in real-world, routine cervical screening, the expert-curated Pap smear image dataset presented here can serve as an important resource for training clinically relevant machine learning models^29^. This dataset prioritizes image quality, reproducible technical preparations, and classification accuracy as critical factors for machine learning workflows.

The Pap smear slides that were used for this dataset were standardized to a single preparation method and automated staining protocol (Hologic ThinPrep®) to minimize technical variability. Image capture and subsequent multi-expert consensus classification was performed using protocols based on clinical best practices. We believe this dataset can be useful to other machine learning researchers who are seeking images that have been rigorously vetted and represent current clinical diagnostic standards in cervical cancer screening.

## Methods

**Fig. 1:**
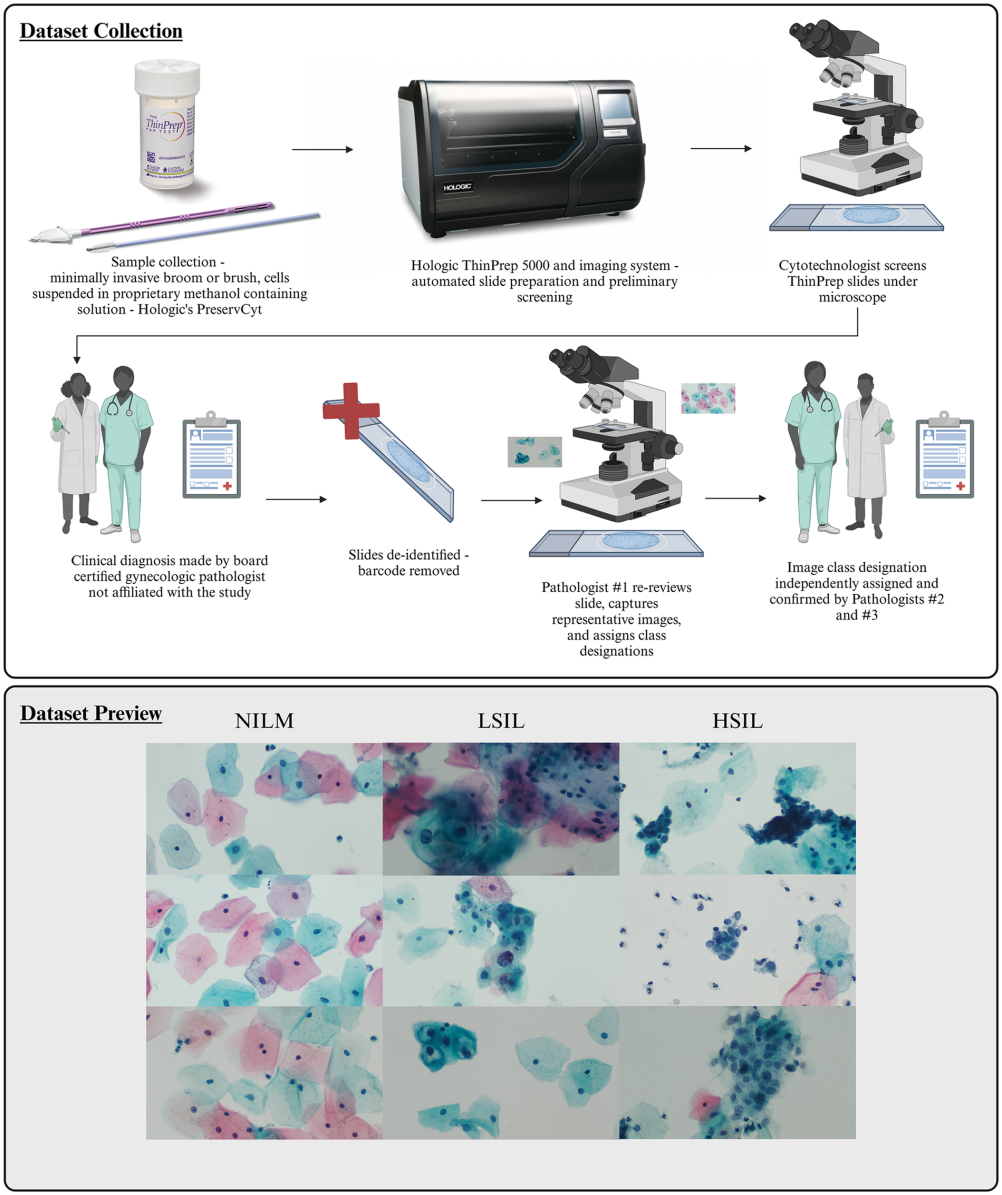
Dataset collection and preview of randomly selected images. The dataset was collected following Hologic’s recommended guidelines for ThinPrep® Pap slide preparation. In particular, the minimally invasive broom and brush methods were used to collect cells from the cervix, which were then placed in Hologic’s PreservCyt solution. Slide preparation was automated using a ThinPrep® 5000 instrument. Slides were initially imaged and a diagnosis was made by 2 expert board-certified cytopathologists who were third parties to this work. The slides were subsequently deidentified by removing the barcode to be used as teaching slides. Another expert board certified cytopathologist then imaged each slide and captured multicellular images, seen in the “Dataset preview” section below. Finally, 2 separate board-certified cytopathologists then reviewed each image. Images were only included if all 3 experts agreed.

This ThinPrep® dataset consists of a balanced set of 600 multicellular images, 200 images per class each of Negative for Intraepithelial Lesion of Malignancy (NILM), Low-Grade Squamous Intraepithelial Lesion (LSIL), and High-Grade Squamous Intraepithelial Lesion (HSIL), which represent clinically relevant diagnostic groups for precancerous cervical cell classification (Fig. 1)^29^. The images have been analyzed independently by three board-certified pathologists to confirm that each image exhibits classic morphologic features that are diagnostic for the designated class.

The images were collected, annotated, and analyzed by academic gynecologic pathologists at Care New England Health System, Women and Infants Hospital of RI (WIHRI), and Brown University. The project was a collaboration between Brown Departments of Pathology and Laboratory Medicine and Biomedical Engineering, with further analytical contributions from the Department of Computer Science.

This collection of 600 images was derived from archival samples and contains no associated or linked patient identifiers. At the time of sample collection, informed consent was given for sample donation for use as educational materials. Due to the absence of patient identifiers, prior research utilizing this dataset was not considered human subjects research and therefore did not require Institutional Review Board (IRB) review. The Brown University IRB was consulted and declared that no formal review was necessary and that open publication of the dataset is permissable.

The following steps were taken in order to construct the database presented herein.

### Data collection

An archival, educational collection of 180 deidentified ThinPrep® Pap slides from 180 patients (WIHRI, Providence, RI) served as the foundation for the images acquired for this dataset. The slides contained no protected health information (PHI) and retained no links to any PHI.

This anonymous educational Pap image dataset was previously obtained for teaching purposes from archival Pap smear sample slides, with no associated protected health information, labels, or identifiers. As part of standard clinical practice, original Pap smear samples were obtained with patient consent by a healthcare provider at the time of medical care. The Institutional Review Board (IRB) was consulted and determined that, in the absence of protected health information, labels, or identifiers, no formal IRB review or waiver was needed, and these anonymous images can be shared in the public domain, permitting open publication of this dataset.

The slides had been previously diagnosed by several pathologists due to their use as training slides. One board-certified gynecologic pathologist examined all previously diagnosed slides microscopically, selected multicellular fields of view for image capture, and assigned a preliminary class designation to each image.

All images (1920 × 1080 pixels) were manually captured using an Olympus BX43 microscope with a 40 X objective, 0.5 X C-mount adaptor and an Excelis HD color microscopy camera. Multicellular field of view (FOV) criteria included: (1) inclusion of at least one diagnostic cell (NILM, LSIL, or HSIL) in the captured image, (2) exclusion of FOV containing both LSIL and HSIL cells, and (3) exclusion (when possible) of potentially “confounding” visual features (such as significant overlapping, debris, mucus or blood). FOV with other types of cellular or equivocal atypia were not included.

At least 1 and a maximum of 5 fields of view images were captured for each of the 180 unique patient slides represented in the dataset, yielding a final total of 600 images with exactly 200 per class.

Due to the biological variability, confounding features cannot be completely avoided in a multicellular image. Since these confounding features may result in diagnostic interobserver variability, an expert consensus vetting protocol was established to validate all images.

Other Bethesda categories such as Atypical squamous cells of undetermined significance (ASCUS) and Squamous Cell Carcinoma (SCC) were not represented in this dataset. In real-world practice, ASCUS can include a significant range of diagnostic interobserver variability, while SCC cases are far less prevalent than NILM, LSIL, and HSIL.

### Dataset validation

After the initial images were captured, two other board-certified gynecologic pathologists classified images into one of three Bethesda classes: NILM, LSIL, or HSIL, according to established diagnostic criteria (Supplementary Table 1). The pathologists were blinded to both the original classification of the slide and the image classification made by the first pathologist.

In order to be included in the final dataset, 100% class consensus was required among all three pathologists. Images where any degree of discrepancy was observed (representing <10% of initial images captured) were not included in the database.

## Data Records

### Repository and dataset format

The “Brown Multicellular ThinPrep” (BMT) dataset described here will be permanently publicly accessible using the following link (10.7303/syn55259257) and released under a Creative Commons Attribution CC BY license^29^. The images can be accessed and downloaded from the given repository. The images themselves are archived in the original JPEG format output from the CaptaVision + Software for Excelis Microscope Cameras (v.2.4.1) and are separated into one of three folders consisting of the validated true class.

**Fig. 2:**
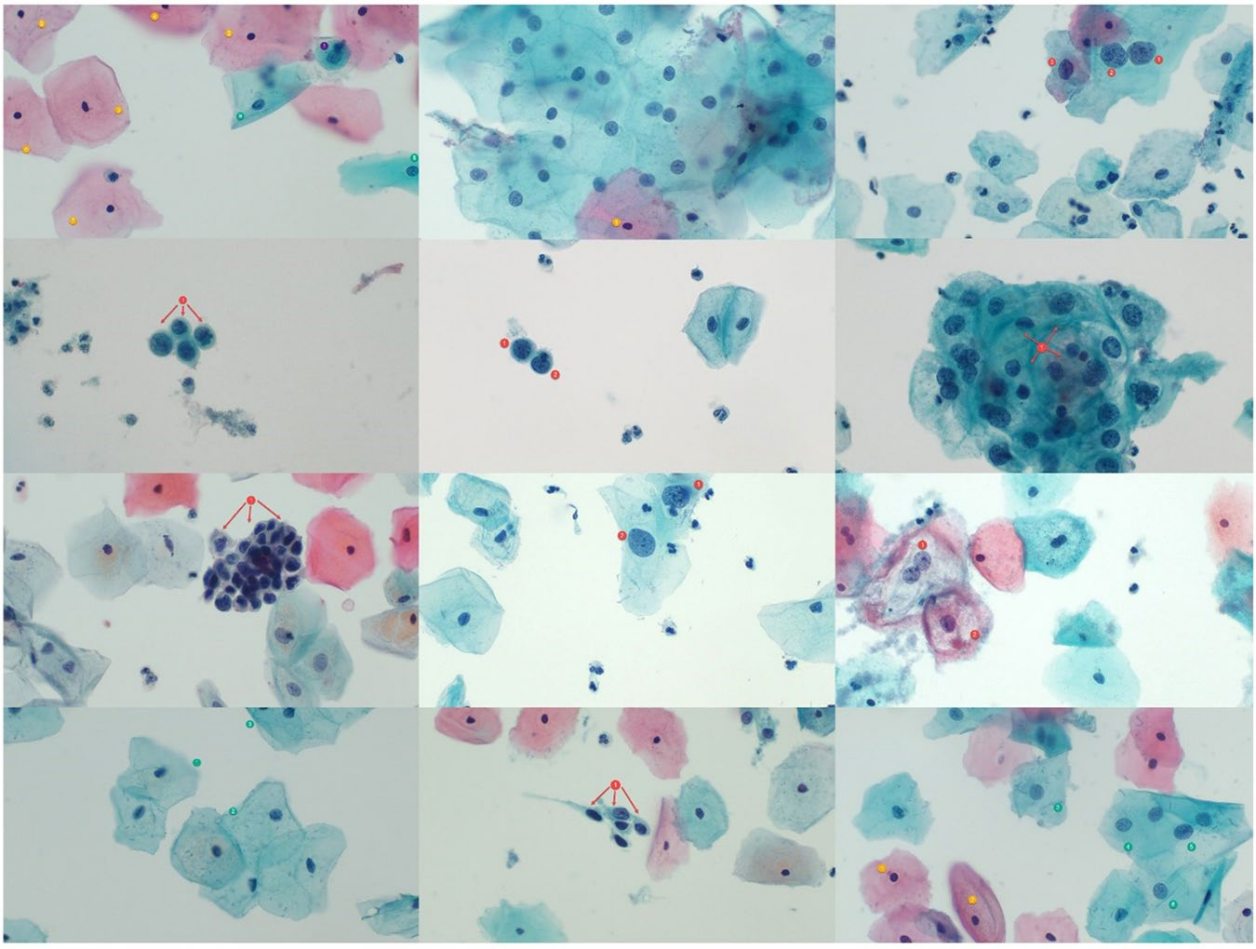
Annotated images from dataset showing important diagnostically relevant features. The full collection of annotated images and specific information on annotation is available on the database page in the “annotated images” subfolder.

The full collection of all images is separated into three categories and included in a sub-folder within the dataset. A separate sub-folder has also been created for annotated images (Fig. 2). These images clearly depict important cellular diagnostic classifications that are relevant in classification of each image. A table has been included in this same sub-folder providing additional context and notes on the classifications of each image.

### Dataset description

180 educational ThinPrep® Pap slides were originally prepared from 180 individual patient cervical samples submitted to WIHRI for diagnosis and clinical reporting. After slide preparation, patient identifiers were removed from the Pap slides prior to their inclusion in the educational collection. At least 1 and a maximum of 5 field of view images were captured from each slide and classified as noted above. The final dataset, with 100% expert consensus, consists of 200 images for each class of NILM, LSIL, and HSIL, for a total of 600 images.

## Technical Validation

### Validation of diagnostic classifications

The diagnostic classifications made were validated against the known slide identities, as described above. Slide identities were originally confirmed with subsequent analyses by other experts, repeat slide collection and analysis, and other tests including HPV tests. Each slide and image region was screened rigorously to ensure that it correctly depicts the class.

### Example usage in machine learning classification

**Fig. 3:**
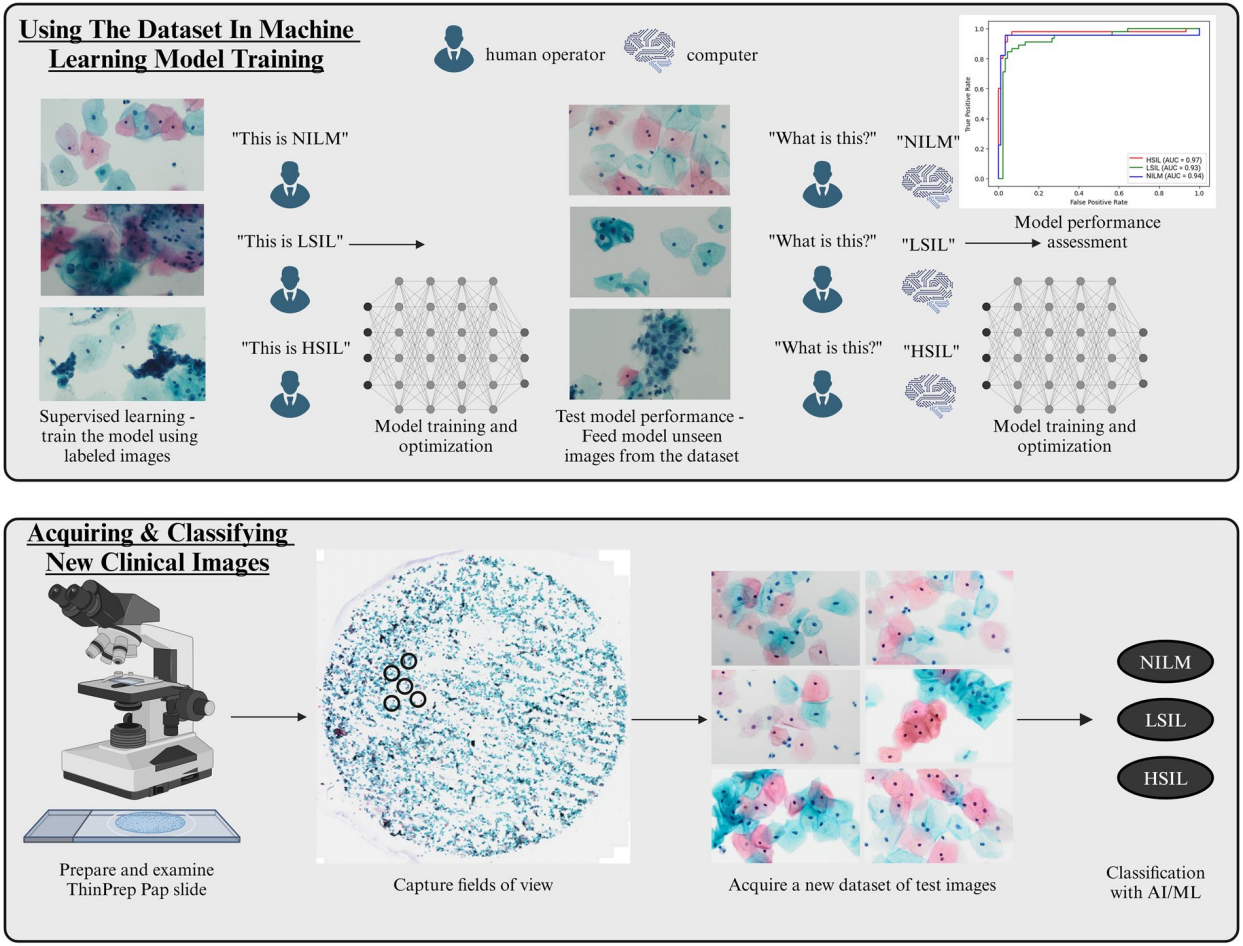
Guidelines for how the dataset can be used in machine learning model training and testing. In order to use the dataset to train a machine learning model, supervised learning can be used, wherein the model will be trained on labeled images. Model performance can then be tested by using new images from this dataset that the model has not yet seen and testing the ability of the model to classify them correctly. Subsequently, the performance of the model can be assessed and further optimized. Once the model is performing as desired, it can be used to classify new clinical images prepared by other researchers and clinicians.

As a proof-of-concept test, the dataset was used to train two basic machine learning models and two convolutional neural network (CNN)-based deep learning models (Fig. 3). First, the dataset was split into 60:20:20 for training, validation, and testing respectively, using a random shuffle method. Data augmentation was conducted on the 360 training images only, using the TensorFlow RanfomFlip and RandomRotation functions. The final augmented dataset consisted of 720 images for the training set, 120 images for validation, and 120 images for testing.

The use of the dataset in training Support Vector Classifier, Random Forest, VGG19, and ResNet50 was analyzed. Data was presented using confusion matrices, classification reports, and receiver operating characteristic (ROC) curves, wherein the area under the curve (AUC) was calculated.

**Fig. 4:**
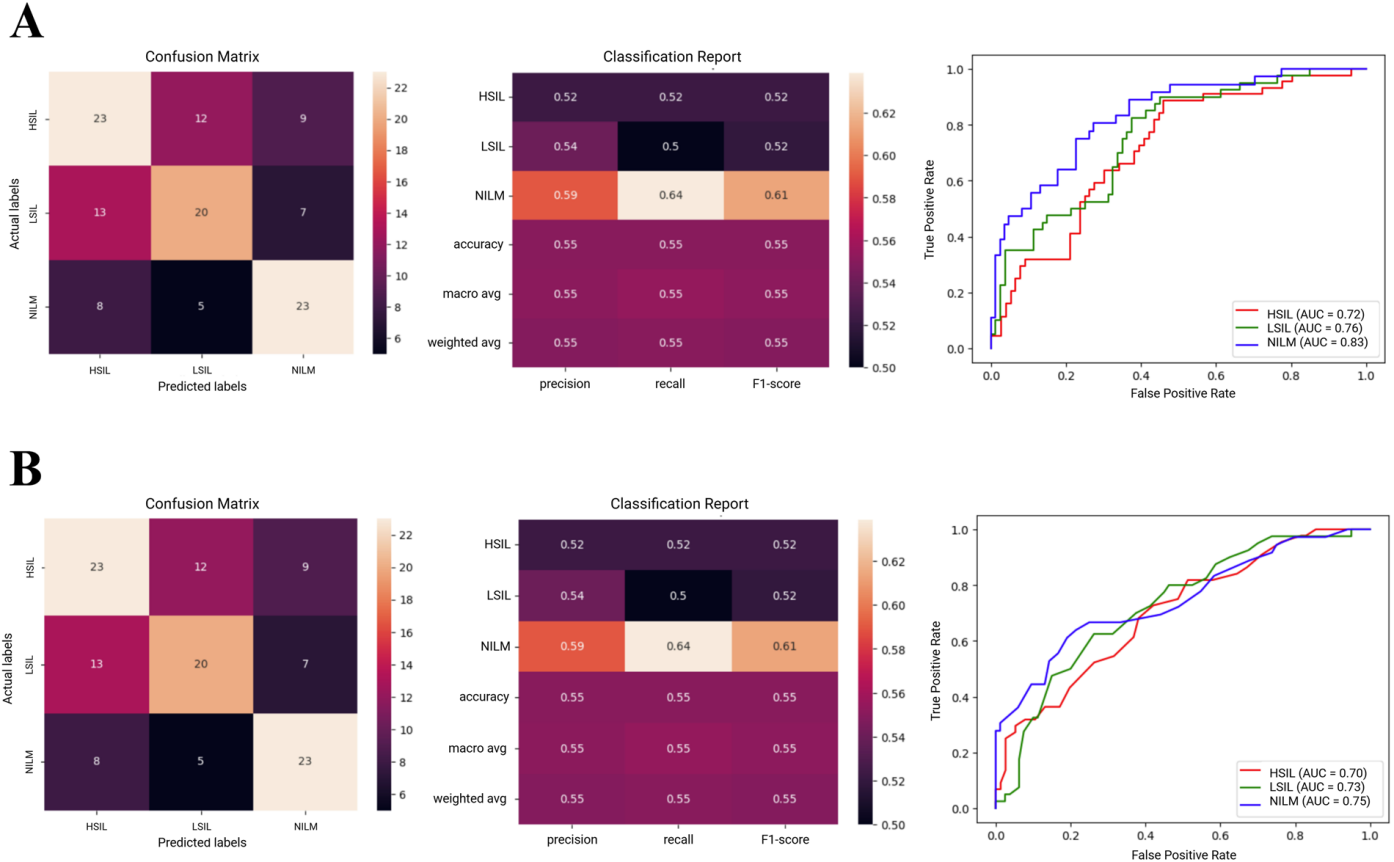
Example results from use of the dataset in basic machine learning model training and image classification tasks. Confusion matrices, classification reports, and Receiver Operating Characteristic curves are provided for (**A**) Support Vector Classifier, and (**B**) Random Forest models.

**Fig. 5:**
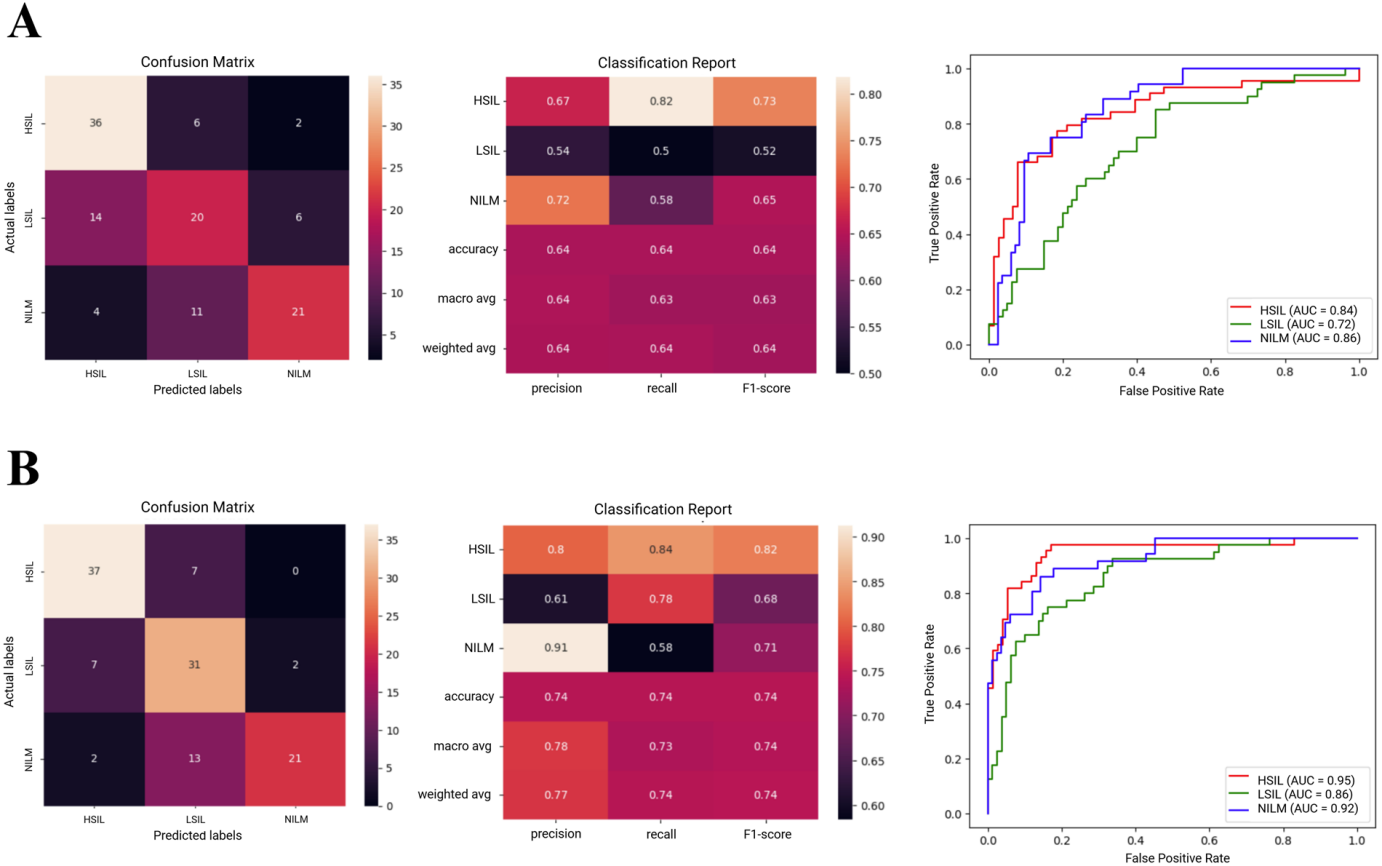
Example results from use of the dataset in training convolutional neural network (CNN) deep learning models for image classification tasks. Confusion matrices, classification reports, and Receiver Operating Characteristic curves are provided for (**A**) VGG19, and (**B**) ResNet50 models.

Accuracies ranged from 55% for the simpler models including Support Vector Classifier and Random Forest (Fig. 4), to 64.16% for VGG19 and 74.16% for ResNet50 (Fig. 5). These results correlate well with our previous findings which showed that deep learning approaches are generally more suitable for this classification task, with ResNet50 performing particularly well in the analysis of unsegmented multicellular liquid Pap images.

**Fig. 6:**
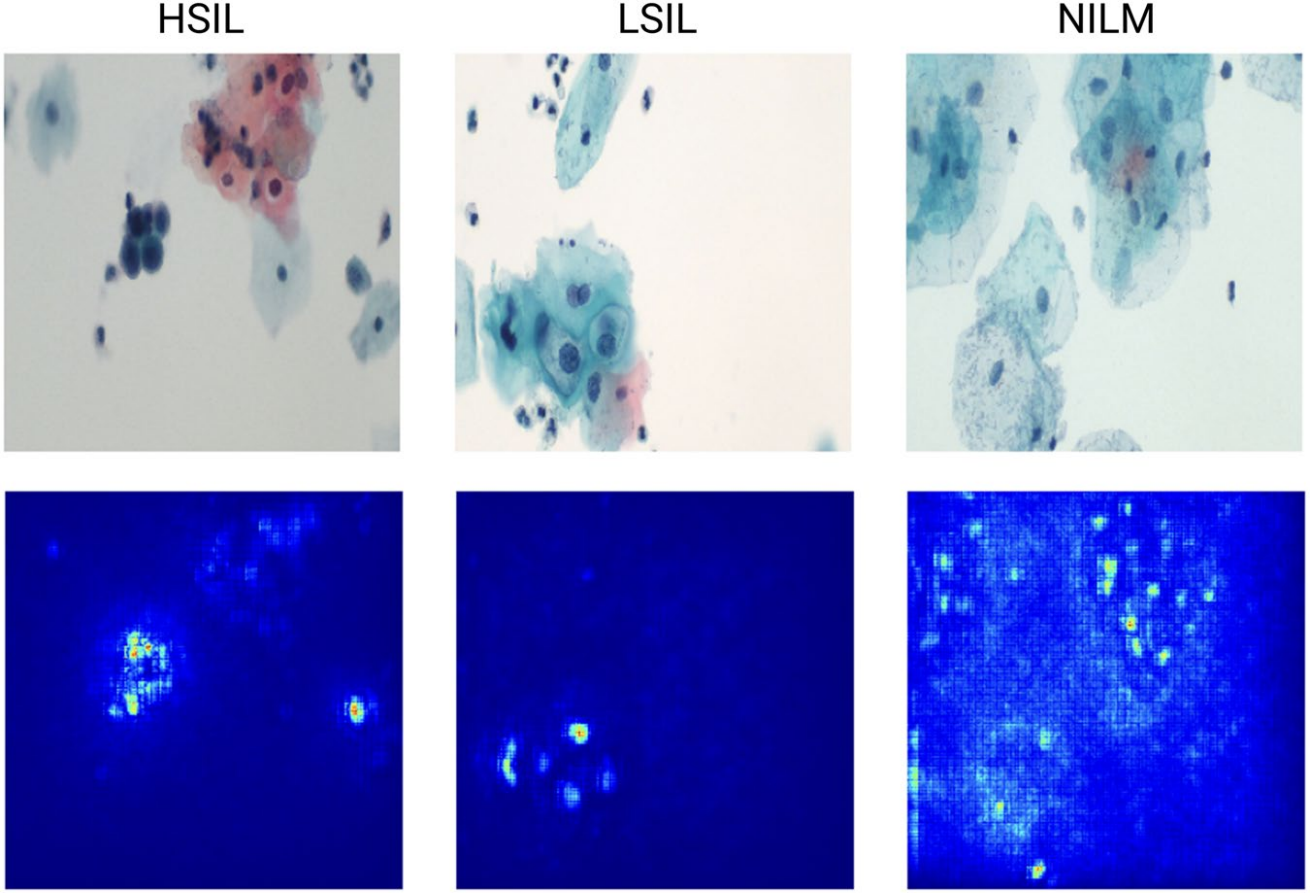
Example saliency results for ResNet50 model showing features of importance in the classification process.

Saliency was analyzed to examine the importance of image features in the classification output to better visualize the decision-making process. Salient object detection maps were then plotted using the Keras data visualization Python package and the Saliency module. These illustrate that certain models, such as ResNet50, are able to focus on cellular features of importance including nuclear morphology (Fig. 6). Simpler ML models will need additional added upstream components including segmentation and feature extraction to better detect relevant features.

While initial performance with basic machine learning models is relatively low, we have produced forthcoming work using this dataset to train and test a hierarchical convolutional neural network (HCNN) model that has achieved accuracy >90%. This accuracy was maintained when making a single classification from testing multiple images at once in different combinations, a process that more closely replicates real-world diagnostic practice. Namely, it is rare that HSIL slides contain only HSIL, they frequently contain LSIL as well. Multi-image classification can thus be used to train models for more complex classification tasks that are more analogous to whole slide imaging workflows. In summary, we expect this dataset to be useful in training and testing modern machine learning models.

## Usage Notes

Our previous work has shown that, to obtain best results, machine learning models must be trained on images that have been collected from slides prepared with the same preparation format as the intended test slides^28^. Even if liquid Pap preparations are used for both the training and test sets, notable differences are observable in cellular morphology when slides are prepared with different liquid Pap methods such as SurePath™ vs ThinPrep®, potentially limiting machine learning model adaptability.

We specifically demonstrated that a model trained on an available SurePath™ Pap multicellular dataset was not able to transfer well to classification of images prepared with the ThinPrep® method, even when using advanced domain adaptation techniques^28^. While >90% accuracy was obtained for every deep learning model trained and tested on other SurePath™ images, accuracies as low as 60.29% were obtained when the SurePath™ trained model was tested on ThinPrep® images, even with domain adaptation.

Increased availability of ThinPrep® Pap multicellular datasets is clinically important as this method becomes increasingly used worldwide. The creation of this resource will enable more robust machine learning model training specific to ThinPrep® images, thereby filling a critical gap in the existing dataset landscape for cervical pathology images. We hope that the publication of this dataset can lead to the construction of larger scale, collaborative, and multi-institutional image sets that can drive machine learning developments to improve cervical cancer diagnosis and prevention.

## Supplementary information

Supplementary Information

## Data Availability

Python scripts for data analysis and comparison are available at https://github.com/celwelch/BMTcode/.
