# Supplementary material for: BMT: A Cross-Validated ThinPrep Pap Cervical Cytology Dataset for Machine Learning Model Training and Validation
Source: Sci Data. 2024 Dec 28;11:1444. doi: 10.1038/s41597-024-04328-3 (PMC11682344; doi:10.1038/s41597-024-04328-3)
Supplement: Supplementary file 1 — Supplementary Information [file 41597_2024_4328_MOESM1_ESM.docx]

**BMT: A Cross-Validated ThinPrep Pap Cervical Cytology Dataset for Machine Learning Model Training and Validation**

**Supplementary Information**

E. Celeste Welch^1^, Chenhao Lu^2^, C. James Sung^2^, Cunxian Zhang^2^, Anubhav Tripathi^1^, Joyce Ou^3^

^1^Center for Biomedical Engineering, School of Engineering, Brown University, Providence, RI 02912, USA

^2^Department of Computer Science, Brown University, Providence, RI 02912, USA

^3^Department of Pathology and Laboratory Medicine, Alpert Medical School, Brown University, Providence, RI 02912, USA

|  | **Cellular Size** | **Cytoplasm** | **Nuclei** | **N:C Ratio** | **Other Notes** |
| --- | --- | --- | --- | --- | --- |
| **SC** | Largest of the normal squamous cells | Translucent, sometimes with keratohyaline granules | Cross-sectional area =10 μm^2^ (average), small round, pyknotic (dark, condensed), often centrally located | 2-3% | Most mature of 3 cell types found in NILM  Most common cell type along with IC |
| **IC** | Slightly smaller than SC | Somewhat translucent | Cross-sectional area =35 μm^2^ (average), oval, with finely granular nuclear chromatin | 3-5% | Second most mature of the 3 cell types found in NILM  IC nuclei are the benchmark for comparison to dysplastic nuclei |
| **PC** | Variable, on average smaller than IC | Rounder, denser, and less translucent cytoplasm than IC | Cross-sectional area =50 μm^2^ (average), round to oval, larger than IC nuclei with finely granular nuclear chromatin | Variable, can be as high as 20% | Least mature of the 3 cell types found in NILM |
| **LSIL** | Variable | Well-defined, can overlap with IC, some cases exhibit perinuclear cytoplasmic halo (clearing) | At least 2-3-fold larger than IC nuclei, generally hyperchromatic, may see: coarse chromatin, irregular nuclear membranes, multinucleation | Variable, greater than that of IC but less than that of HSIL | Cells can be found as single cells or in clusters |
| **HSIL** | Variable, often smaller than LSIL cells | Variable volume and texture | Variable sizing (may be larger or smaller than LSIL nuclei), variable texture, may see significantly irregular nuclear membranes, chromatin can be fine or coarse | Usually markedly increased | Cells can be found as single cells, sheets, or syncytial aggregates |

Supplementary Table 1 - Characteristics of different cell types used in classification of precancerous cervical cell abnormalities. Superficial squamous cells (SC), intermediate squamous cells (IC), and parabasal cells (PC) cell types may be seen in any Pap samples and are considered normal (i.e. NILM). LSIL and HSIL categories include cells with features of low and high grade dysplasia, respectively, also referred to as low-grade and high-grade intraepithelial lesions. (Adapted from The Bethesda System for Reporting Cervical Cytology, 3rd Edition, 2015).
